# Low value of detection of KRAS2 mutations in circulating DNA to differentiate chronic pancreatitis to pancreatic cancer

**DOI:** 10.1038/sj.bjc.6601854

**Published:** 2004-05-04

**Authors:** R Marchese, A Muleti, S Brozzetti, O Gandini, E Brunetti, D French

**Affiliations:** 1 AFaR S Pietro Hospital Research Centre, Rome, Italy; 2 Department of Surgery, University of Rome, “La Sapienza”, Rome, Italy; 3 Department of Sperimental Medicine and Pathology, University of Rome, “La Sapienza”, Rome, Italy; 4 S. Andrea Hospital, Molecular Pathology Unit, 2^nd^ Medicine Faculty of University of Rome, “La Sapienza”, Rome, Italy

**Sir**,

We read with great interest the article by Maire *et al* (2002), who evaluate the K-Ras mutations in circulating DNA to differentiate pancreatic cancer from chronic pancreatitis. Based on this, we also analysed KRAS2 mutations in the serum of 30 patients with pancreatic cancer and 40 patients with chronic pancreatitis. Pancreatic cancer patients were staged by means of dynamic computed tomography, magnetic resonance imaging, and angiography and/or endoscopic ultrasonography. Diagnosis was histologically confirmed for the patients who underwent surgery.

The diagnosis of chronic pancreatitis was based on the radiologic data obtained by means of either endoscopic retrograde cholangiopancreatography or computed tomography.

DNA was extracted from 20 ml of the serum by using the QIAmp Blood Kit (Qiagen) and the mutations in codon 12 of the K-ras gene were searched as described previously (Jiang *et al*, 1989).

As positive controls, we used DNA from neoplastic tissues of 10 patients with pancreatic carcinoma by using the DNeasy Tissue Kit (Qiagen). For molecular analysis, DNA was amplified in the codon 12 region introducing a restriction site (GACCT) for digestion with *Bst*Nl restriction enzyme (PCR-RFLP).

DNA from peripheral blood resulted not mutated in the 40 patients with chronic pancreatitis and in the 30 with pancreatic carcinoma, while DNA from pancreatic neoplastic tissue resulted mutated in 70% of the samples.

To verify our results, all the samples were analysed by direct sequencing using Big Dye terminator v 1.1 cycle sequencing Kit and performing runs on ABI Prism 310 genetic analyzer (Applied Biosystem)

Despite what was mentioned in Maire's article, we failed to find any mutations in all patients analysed, as well as we failed to correlate K-ras mutations with the levels of tumour markers such as Ca 19.9, CA242, CA50, CEA.

The results of the present investigation lead us to these conclusions: (1) the eventual presence of cancer cells in peripheral blood may be a rare event, even if numerous reports support the detection of K-ras abnormalities in the serum, (2) neoplastic cells are supposed to circulate in clusters, and consequently their cognition could be hampered by a single blood sample extraction. (3) Large amounts of nonmutated DNA, coming from leucocytes held in the buffy coat layer, might also mask some vestiges of the mutant type of K-ras gene.
